# Double Trouble: An Uncommon Case of Ocular Toxoplasmosis in a Systemic Lupus Erythematosus (SLE) Patient

**DOI:** 10.7759/cureus.110402

**Published:** 2026-06-07

**Authors:** Manu Priya, Supriya Rawat, Kanavdeep Kapoor, Shrutanjoy Das, Vaibhav Bhatt, Gaurav Luthra, Subodh Gururani, Saurabh Luthra

**Affiliations:** 1 Ophthalmology, Drishti Eye Institute, Dehradun, IND; 2 Rheumatology, Shri Guru Ram Rai Institute of Medical and Health Sciences (SGRRIMHS) and Shri Mahant Indiresh Hospital (SMIH), Dehradun, IND

**Keywords:** immune dysregulation, ocular toxoplasmosis, opportunistic infection, retinochoroiditis, systemic lupus erythematosus

## Abstract

Systemic lupus erythematosus (SLE) is a chronic autoimmune condition characterized by immune dysregulation and use of immunosuppressive therapy, predisposing patients to opportunistic infections. Although infections are a major cause of morbidity and mortality in SLE, ocular toxoplasmosis is rarely reported in these patients and may pose a diagnostic challenge due to its ability to mimic other inflammatory or infectious ocular conditions. Early recognition is essential for timely management and improved visual outcomes.

Here we report the case of a 21-year-old woman, a known case of SLE on maintenance immunomodulatory therapy, who presented with a blurring of vision in the right eye since 15 days. Ocular examination revealed vitritis with an active focus of necrotizing retinochoroiditis. Multimodal imaging, including ultrawide field fundus photography, fundus autofluorescence, and spectral domain optical coherence tomography, supported the clinical diagnosis of ocular toxoplasmosis. Serological evaluation showed positive immunoglobulin G (IgG) for *Toxoplasma gondii*, with negative IgM. The patient was treated with oral trimethoprim-sulfamethoxazole and corticosteroids, resulting in a progressive clinical improvement and resolution of the lesion with scarring. During follow-up, the patient also developed an SLE flare and herpes zoster infection, highlighting the complexity of persistent immune dysregulation in such patients, despite apparent clinical stability.

Although uncommon, ocular toxoplasmosis should be considered in the differential diagnosis of posterior uveitis in SLE patients. Clinical examination supported by multimodal imaging remains crucial for diagnosis, particularly when serological tests are inconclusive. Prompt diagnosis and appropriate therapy can lead to favourable visual outcomes and prevent potentially life-threatening systemic complications.

## Introduction

Systemic lupus erythematosus (SLE) is an autoimmune disorder marked by chronic overactivation of the immune system, resulting in inflammation [1]. It can present with a wide variety of clinical manifestations ranging from mild cutaneous involvement to severe multiorgan dysfunction [2]. Ocular manifestations are common in SLE, affecting about one-third of the patients [2,3]. Women are most commonly diagnosed with SLE, with typical onset occurring between 20 and 30 years of age [4]. In children, SLE follows a more severe course than in adults, with a higher frequency of malar rashes, nephritis, pericarditis, hematologic abnormalities, and hepatosplenomegaly [5]. Despite the availability of several effective therapeutic agents, the disease continues to be associated with considerable morbidity and mortality.

Patients with SLE are at a substantial risk of infections due to immune dysregulation related to the disease itself as well as the use of immunosuppressive therapy [6]. Previous studies have reported that nearly 50% of patients experience at least one serious infection during the disease course, with infections accounting for a significant proportion of hospital admissions and mortality [7-9]. The majority of the infections in SLE patients are bacterial in origin [10]. Irrespective of the use of immunosuppressants, the most frequently encountered infections include *Streptococcus pneumoniae*, *Escherichia coli*, and *Staphylococcus aureus* [11,12]. Additionally, due to impaired cell-mediated immunity, viral and fungal infections may occur concurrently. Other commonly reported infections comprise hepatitis B virus (HBV), hepatitis C virus (HCV), tuberculosis, herpes zoster and *Pneumocystis jiroveci* pneumonia (PJP) [13].

Toxoplasmosis results from an infection with *Toxoplasma gondii*, which can occur through congenital or acquired means [14]. It is estimated that nearly one-third of the global population is chronically infected with *T. gondii* [15,16]. The majority of the affected individuals do not exhibit any symptoms [17]. Toxoplasmosis is an opportunistic infection commonly occurring in immunocompromised states such as acquired immunodeficiency syndrome (AIDS), malignancy, and use of chronic immunosuppressive drug therapy in organ transplant recipients. In contrast, toxoplasmosis is not considered a frequent opportunistic infection affecting patients with autoimmune diseases receiving immunosuppressive treatment [18].

Opportunistic infections usually present with localized disease, milder inflammation, slower progression, and more typical clinical features in immunocompetent individuals due to relatively preserved immune control. In contrast, immunosuppressed patients often develop atypical, multifocal, rapidly progressive, or disseminated disease with higher pathogen load, reduced inflammatory response, increased tissue destruction, and poorer outcomes [19,20]. Ocular toxoplasmosis in immunocompetent individuals classically presents as a unilateral focal necrotizing retinochoroiditis adjacent to a pre-existing pigmented chorioretinal scar, often associated with dense vitritis, producing the characteristic “headlight in the fog” appearance [21]. In contrast, in immunocompromised patients, the disease may manifest atypically with larger or multifocal retinal lesions, bilateral involvement, absence of an adjacent old scar, and relatively minimal vitritis despite extensive retinal necrosis [22]. This altered clinical phenotype is attributed to impaired cellular immunity, which permits unchecked proliferation of the parasite and may result in a more fulminant course. Consequently, management in immunocompromised hosts is generally more aggressive, often requiring early initiation of systemic antiparasitic therapy, prolonged treatment duration, and close monitoring for recurrence, unlike in immunocompetent individuals, in whom treatment may be individualized based on lesion location and severity [22,23].

The objective of this report is to describe a rare case of ocular toxoplasmosis in an SLE patient and to emphasize the role of multimodal imaging in establishing an early diagnosis in the presence of overlapping infectious and autoimmune manifestations. Given that outcomes are strongly influenced by early recognition and appropriate therapy, it is crucial for both ophthalmologists and rheumatologists to be familiar with its manifestations and to actively consider this diagnosis in such patients.

## Case presentation

A 21-year-old woman reported to our hospital in July 2025, with the complaint of blurring of vision in the right eye (OD) since 15 days, which was insidious in onset and painless. There was no history of any ocular complaints in the past.

She consulted a local doctor one week prior, where she was diagnosed as OD anterior uveitis and the findings were noted as OD keratic precipitates (KPs) and anterior chamber (AC) cells 4+. She was investigated at the primary centre (Table 1): Cytomegalovirus (CMV) immunoglobulin G (IgG) at 1421.9 AU/mL (reference range <12.0 AU/mL) and immunoglobulin M (IgM) at 0.45 U/mL (reference range <0.85 U/mL), herpes simplex virus (HSV) 1 & 2 IgG at 1.03 AU/mL (reference range <2.0 AU/mL) and IgM 0.02 AU/mL (reference range <2.0 AU/mL), Rubella IgG at 641.2 IU/mL (reference range <5.0 IU/mL) and IgM at 0.11 AU/mL (reference range <1.2 AU/mL), Toxoplasma IgG at >200.0 IU/mL (reference range <1.6 IU/mL) and IgM at 0.42 AU/mL (reference range <9.5 AU/mL). Venereal Disease Research Laboratory (VDRL) test for syphilis, human immunodeficiency virus (HIV) 1 and 2, hepatitis B surface antigen (HBsAg) and anti-hepatitis C virus (anti-HCV) were non-reactive (NR). Complete blood count (CBC), liver function test (LFT), kidney function test (KFT), lipid profile, erythrocyte sedimentation rate (ESR), and C-reactive protein (CRP) were all within normal limits. At presentation to our hospital, she was using eye drop (E/D) moxifloxacin 0.5% + prednisolone 1% four times a day, E/D nepafenac 0.1% three times a day and E/D atropine 1% three times a day in OD.

**Table 1:** Enzyme-linked Immunosorbent Assay (ELISA) test for TORCH (Toxoplasmosis, Other, Rubella, Cytomegalovirus, Herpes simplex virus) done at the primary centre CMV: Cytomegalo virus, IgG: Immunoglobulin G, IgM: Immunoglobulin M, HSV: herpes simplex virus.

Investigation	Result	Reference Range
CMV IgG	1421.9 AU/mL	<12.0 AU/mL
CMV IgM	0.45	<0.85
HSV 1 and 2 IgG	1.03 AU/mL	<2.0 AU/mL
HSV 1 and 2 IgM	0.02 AU/mL	<2.0 AU/mL
Rubella IgG	641.2 IU/mL	<5.0 IU/mL
Rubella IgM	0.11	<1.2
Toxoplasma IgG	>200.0 IU/mL	<1.6 IU/mL
Toxoplasma IgM	0.42	<9.5

Her past medical history showed an episode of acute severe drug-induced liver injury in 2020, which was managed conservatively. She was also a known case of SLE since January 2021. She had initially presented to the rheumatologist with generalized alopecia, malar rash, palatal ulcers, vasculitis rash on palms and soles and healed acute cutaneous lupus erythematosus (ACLE). When investigated, she was found to have a positive serum antinuclear antibody (1:100 primary dilution, 2+ primary intensity on immunofluorescence on human epithelial type 2 {Hep-2} cells with nuclear fine speckled pattern, and 1:320 end-point titre). C3 Complement 49.20 mg/dL (reference range 90.00-180.00 mg/dL) and C4 Complement <8.00 mg/dL (reference range 10.00-40.00 mg/dL) were low and anti-double-stranded deoxyribonucleic acid antibody (anti-ds DNA antibody) at 839.06 IU/mL (reference range <30.00 IU/mL) and lactate dehydrogenase at 342 U/L (135-225 U/L) were elevated. Ultrasound abdomen and pelvis revealed borderline hepatosplenomegaly, while the remaining investigations, including CBC, KFT, CRP, ESR and echocardiography (ECHO) were normal. A diagnosis of juvenile SLE-acute cutaneous lupus erythematosus (jSLE-ACLE) was made, and the patient was started on tablet (Tab.) prednisolone 15 mg once a day, Tab. hydroxychloroquine (HCQs) 200 mg once a day, Tab. mycophenolate mofetil (MMF) 500 mg once a day, Tab. ranitidine 150 mg twice a day, Tab. calcium+vitamin D3 twice a day and sunscreen with sun protection factor (SPF)>30 application locally. Tab. MMF (Mycophenolate Mofetil) was gradually increased over the subsequent month to 1000 mg twice daily and then gradually tapered over the following two-and-a-half years and eventually stopped. At presentation, the patient was on Tab. hydroxychloroquines (HCQs) 200 mg on alternate days. There was no history of any sexual activity or any other serious infections in the past.

**Figure 1:**
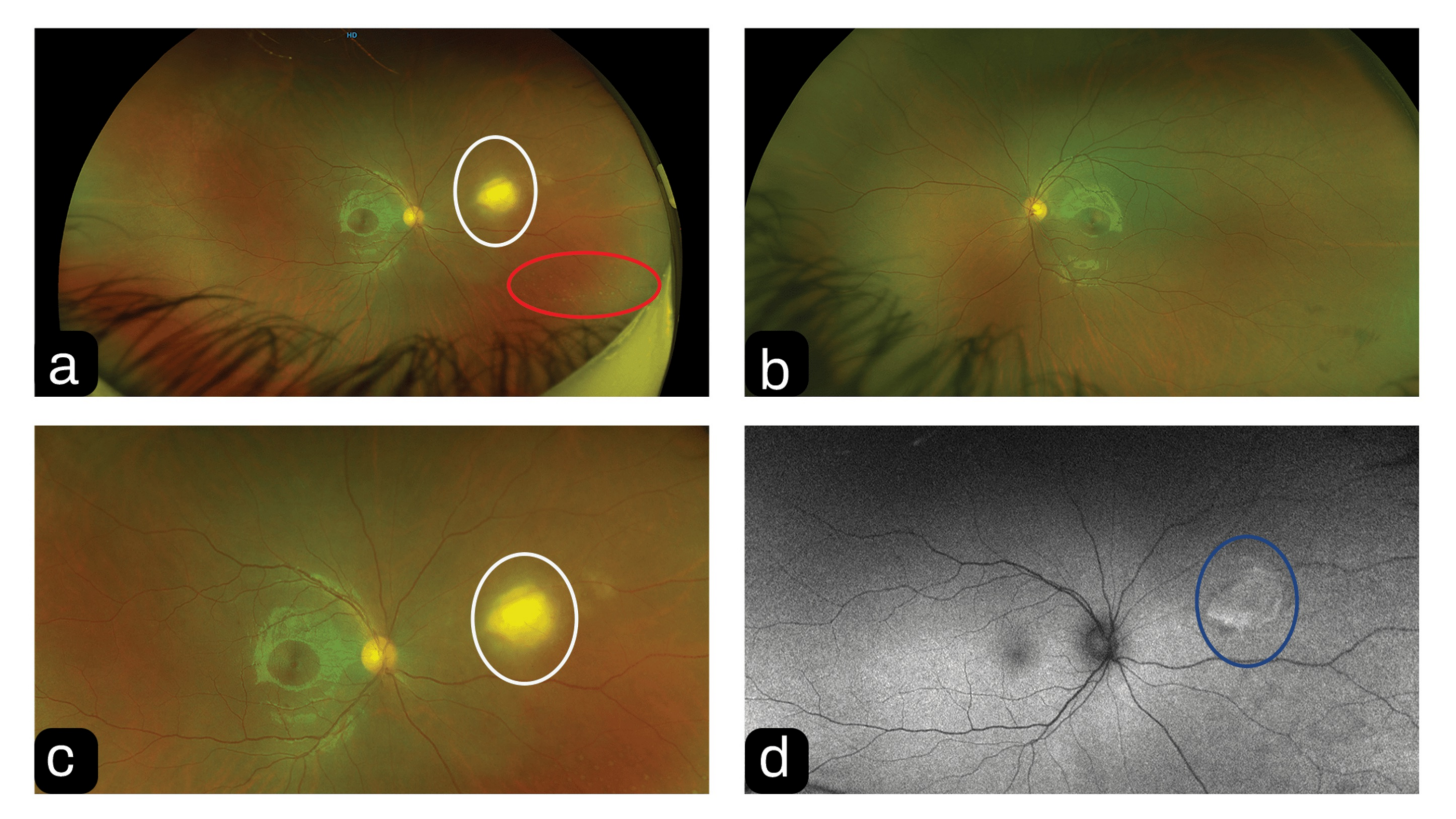
Ultra-wide field (UWF) imaging at presentation (a, c) UWF imaging showing OD active yellowish retinochoroiditis lesion (white circle) present nasal to the disc with fuzzy margins, along with hyperreflective spherical deposits (red circle) within the vitreoretinal interface. (b) OS UWF showing normal fundus. (d) Fundus autofluorescence (FAF), showing stippled hyper + hypo autofluorescence with irregular margins in the area corresponding to the lesion (blue circle). UWF: Ultra-wide field; OD: right eye; OS: left eye

On examination, best corrected visual acuity (BCVA) in OD was 6/24 and left eye (OS) was 6/6. Intraocular pressure (IOP) was normal in both eyes. Anterior segment examination of both eyes was unremarkable (quiet anterior chamber). On ultra-wide field fundus evaluation (Daytona Optos®, Dunfermline, Scotland, UK), vitritis grade 1 was present in OD. There was an active yellowish retinochoroiditis lesion present nasal to the disc with fuzzy margins (Figure 1 a,c). Inferonasal to this patch, there was an overlying thickening of posterior hyaloid along with hyperreflective spherical deposits within the vitreoretinal interface (Figure 1a). Fundus examination of OS was within normal limits (Figure 1b). On fundus autofluorescence (FAF), there was stippled hyper + hypo autofluorescence in the area of the lesion, with irregular margins (Figure 1d).

**Figure 2:**
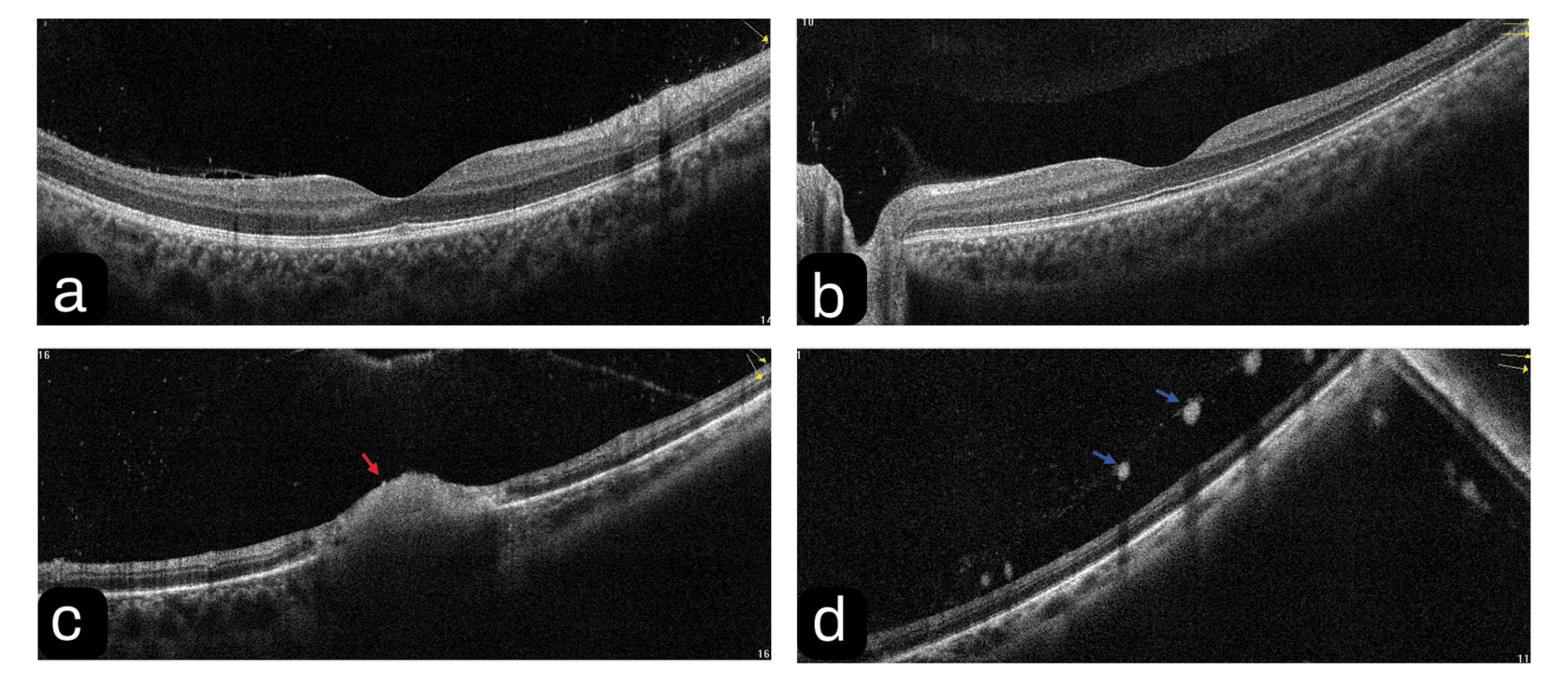
Spectral Domain Optical Coherence Tomography at presentation SD-OCT showing normal foveal contour in (a) OD, (b) OS, (c) SD-OCT through the lesion nasal to the disc in OD, showing localized elevated retinal thickening (red arrow) with disruption of retinal and choroidal architecture. (d) Hyperreflective round deposits (blue arrows) along the posterior hyaloid inferonasal to the lesion in OD. SD-OCT: Spectral domain optical coherence tomography, OD: right eye, OS: left eye.

Spectral domain optical coherence tomography (SD-OCT) (RTVue XR Avanti, Optovue Inc., Fremont, CA) through the macular region was normal (Figure 2a,b). SD-OCT through the lesion showed localized elevated hyperreflective retinal thickening with disruption of retinal and choroidal architecture (Figure 2c), along with hyperreflective round deposits along the posterior hyaloid (Figure 2d). Based on the classic clinical picture, a diagnosis of OD toxoplasma retinochoroiditis with vitritis was made. The patient was started on Tab. trimethoprim 160 mg + Tab. sulfamethoxazole 800 mg twice a day under physician supervision along with E/D prednisolone six times a day (slow tapering dose) and E/D moxifloxacin four times a day and was advised to review with the rheumatologist, where Tab. prednisolone 7.5 mg once a day was added.

**Figure 3:**
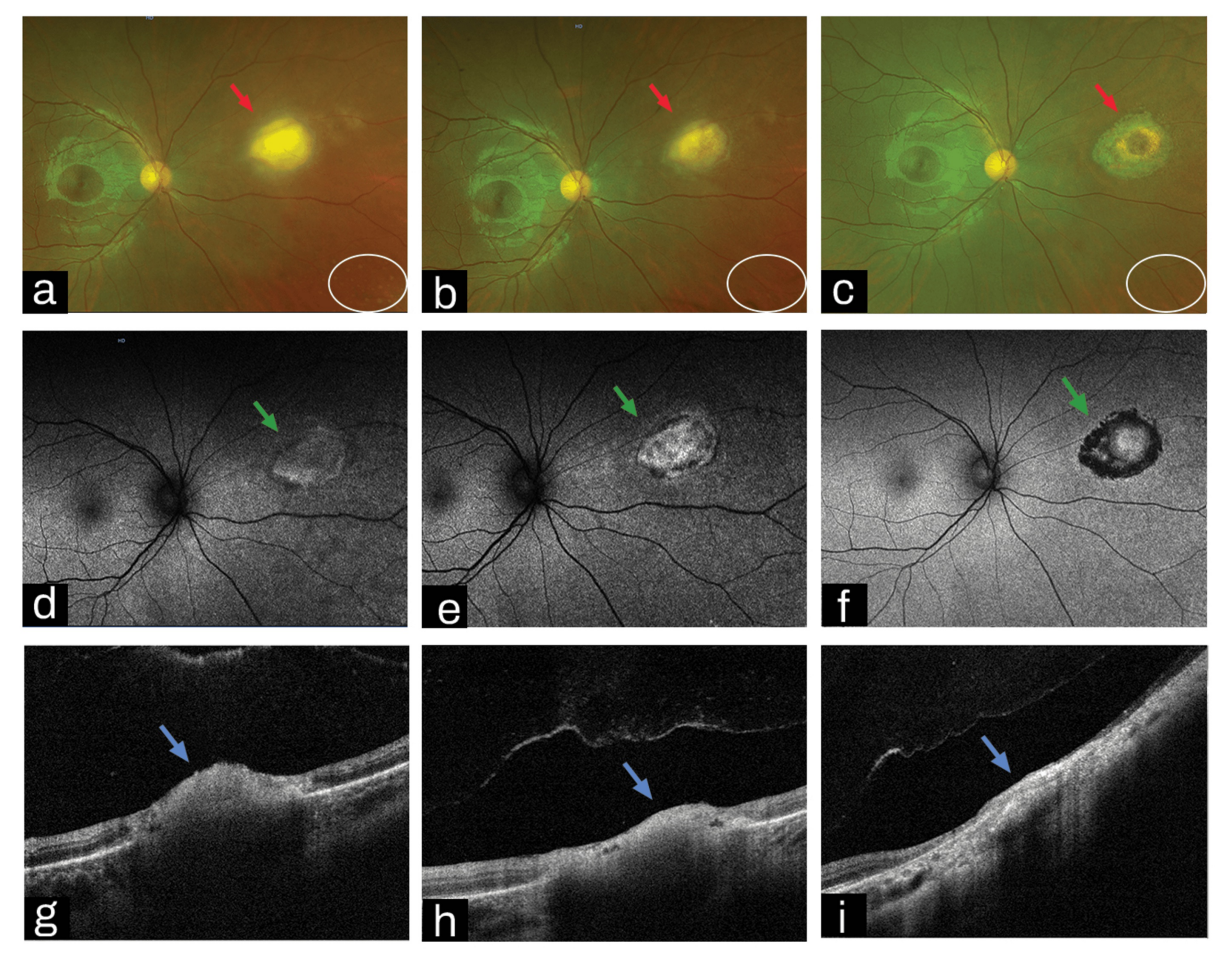
Multimodal imaging at sequential follow up (initial presentation, two weeks follow-up and six months follow-up) (a-c): UWF Optos fundus image showing the lesion turning from bright yellow with fuzzy margins to a more subtle yellow with some scarring and well-defined margins (red arrows), along with resolution of the hyperreflective deposits present within the vitreoretinal interface (white circle). (d-f): FAF image showing initial stippled hypo+hyperautofluorescence at presentation, FAF images at follow-up revealing an increase in hypoautoflourescence at the borders with areas of hyperautofluorescence in the centre, depicting progressive healing of the lesion (green arrows). (g-i): SD-OCT image through the lesion (blue arrows) showing progressive flattening of the lesion and increase in scarring. UWF: Ultra-wide field; FAF: fundus autofluorescence; SD-OCT: spectral domain-optical coherence tomography.

The patient’s sequential follow-up was documented using multimodal imaging (Figure 3a-i). At two weeks follow-up, the BCVA in OD had improved to 6/9. The retinochoroiditis lesion was in the resolving stage and the margins had become less fuzzy with some degree of scarring in the nasal aspect (Figure 3b,e,h). At two months follow-up, BCVA in OD had improved to 6/6P and the lesion was in the resolving stage with distinct margins, peripheral scarring with some degree of activity in the centre. There was also a decrease in the hyperreflective deposits that were present within the vitreoretinal interface. FAF showed hypoautofluorescent borders and stippled hyper+hypo autofluorescence in the centre. The SD-OCT picture showed flattening of the lesion with a decrease in the hyperreflective thickening and disappearance of the posterior hyaloid deposits.

**Figure 4:**
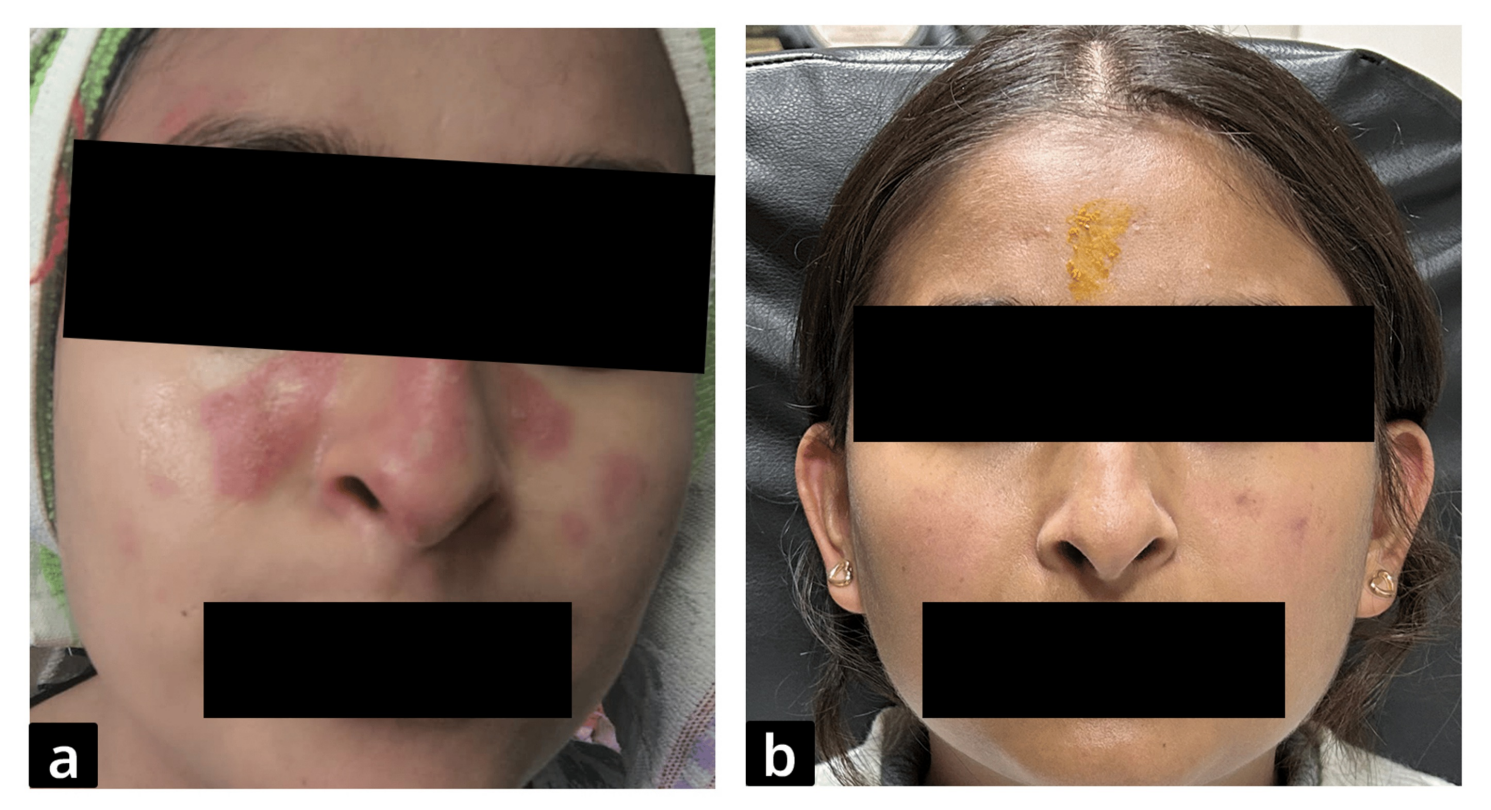
Episode of systemic SLE flare up and subsequent resolution a: Flare up of malar rash at two months b: Flare subsided after increasing steroid dose and adding Tab. methotrexate SLE: Systemic lupus erythematosus.

However, at two months follow-up, the patient had a flare-up of her systemic SLE symptoms in the form of malar rash and palatal ulcers (Figure 4a). She consulted her rheumatologist and Tab. HCQs 200 mg was advised once daily, Tab. prednisolone was stepped up to 30 mg once a day (gradual tapering dose) and Tab. methotrexate 15 mg once a week, Tab. folic acid 5 mg once a week along with calcium and vitamin D supplements were added. The malar rash and palatal ulcers gradually resolved (Figure 4b).

**Figure 5:**
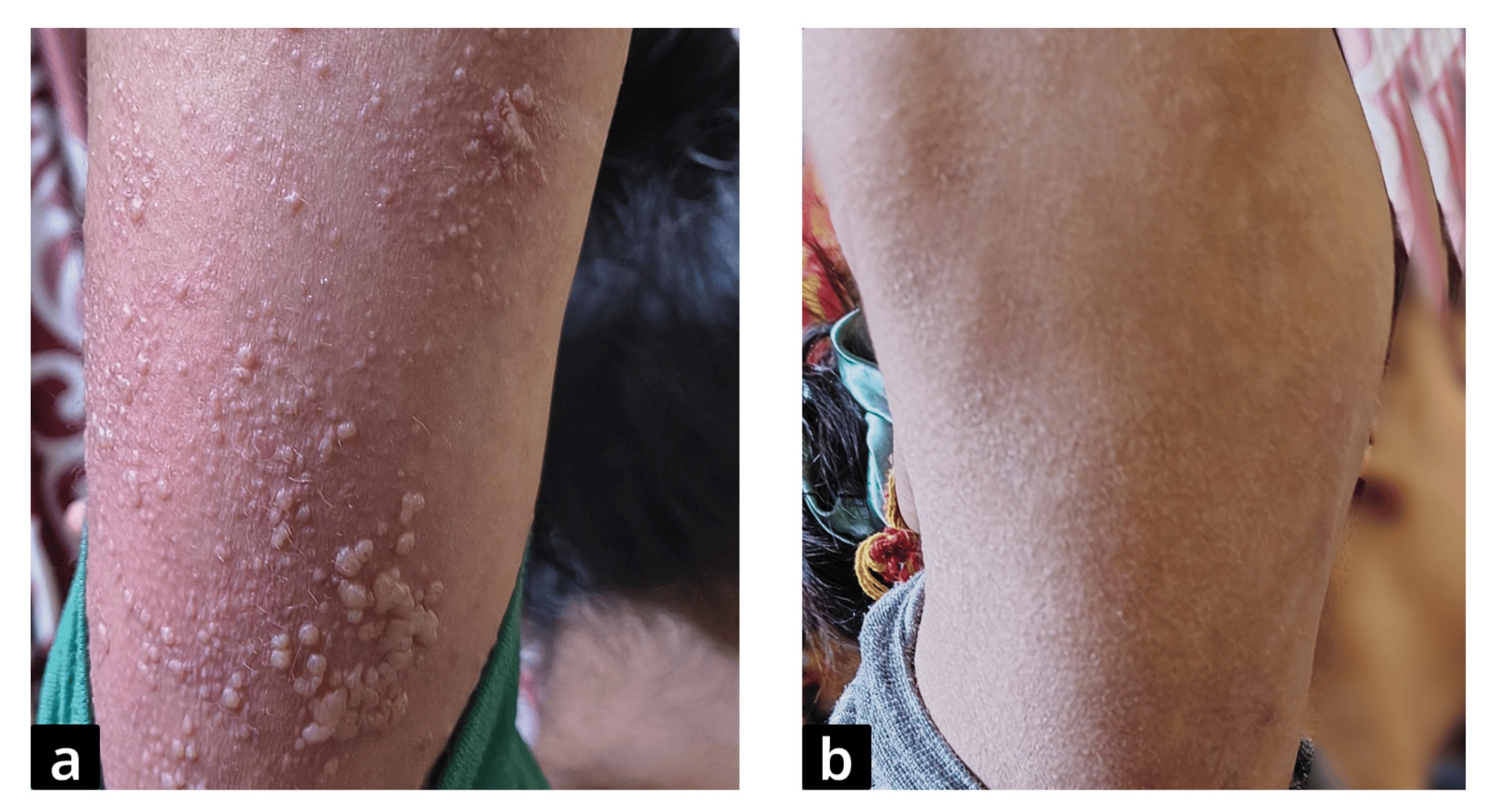
Episode of herpes zoster a: At five months follow-up from initial presentation to us. b: Four weeks after starting anti-herpes treatment showing resolving lesions.

At five months from her initial presentation to us, the patient developed a rash over her back and arms (Figure 5a), for which she consulted a dermatologist and based on the classic clinical presentation, was diagnosed with herpes zoster. The patient was started on Tab. valaciclovir 1 gm thrice a day, cream acyclovir 5% twice a day and Tab. aceclofenac (100 mg) and Tab. paracetamol (325 mg) as needed. At the latest follow-up (six months after initial presentation), BCVA in OD was 6/6P and the lesion showed progressive scarring, which correlated with the FAF and SD-OCT findings (Figure 3 c, f, i). Her prednisolone dose was lowered to 7.5 mg once daily. The rash on her back and arms had resolved (Figure 5b) and her herpes zoster treatment was stopped. The patient is currently under the close supervision of a rheumatologist, a dermatologist and an ophthalmologist.

Table 2 shows a concise tabulated summary of the key clinical parameters on sequential follow-up.

**Table 2:** Tabulated summary of the key clinical parameters on sequential follow up BCVA: Best-corrected visual acuity, FAF: fundus autofluorescence, SD-OCT: spectral domain-optical coherence tomography.

	At Presentation	2 weeks	2 months	6 months
BCVA	6/24	6/9	6/6P	6/6P
Fundus findings	Bright yellow retinochoroiditis lesion with fuzzy margins, hyperreflective spherical deposits within the vitreoretinal interface	Lesion with less fuzzy margins with some degree of scarring in the nasal aspect, decrease in the hyperreflective spherical deposits within the vitreoretinal interface	Lesion with distinct margins, peripheral scarring with some degree of activity in the centre, decrease in the hyperreflective deposits within the vitreoretinal interface	Subtle yellow lesion with scarring and well defined margins, resolution of the hyperreflective deposits within the vitreoretinal interface
FAF	Stippled hyper+hypo autofluorescence with irregular margins	Increase in hypoautoflourescence at the borders with some areas of hyper + hypo autofluorescence in the centre	Hypoautofluorescent borders and stippled hyper+hypo autofluorescence in the centre	Hypoautofluorescent lesion with well-defined margins
SD-OCT	Localized elevated hyperreflective retinal thickening, disruption of retinal and choroidal architecture, hyperreflective round deposits along the posterior hyaloid	Slight flattening of the lesion and slight increase in scarring, decrease in hyperreflective round deposits along the posterior hyaloid	Progressive flattening of the lesion, disappearance of the posterior hyaloid deposits	Flat lesion with scarring and retinal thinning

## Discussion

Toxoplasmosis usually has been reported in severely immunocompromised or immunodeficient patients such as patients with HIV or after organ transplantation. Only a handful cases of toxoplasmosis in SLE patients have been reported in the literature. Most of these infections affected the brain and were only diagnosed on autopsy after death [18,24-27]. To the best of our knowledge, only four cases of ocular toxoplasmosis in SLE patients have been reported till now (Table 2) [18,28-30]. Most of those cases were initially misdiagnosed as acute retinal necrosis, SLE-related retinochoroiditis and neuropsychiatric SLE and started on treatment accordingly. Only after initial treatment failure and more thorough investigations, a diagnosis of ocular toxoplasmosis was made, which led to a delay in the initiation of proper management. After treatment for toxoplasmosis was started, all the patients showed gradual improvement.

**Table 3:** Previously reported cases of ocular toxoplasmosis in SLE patients ARN: Acute retinal necrosis; PCR: polymerase chain reaction; *T. gondii:* *Toxoplasma gondii*; CMV: cytomegalovirus; SLE: systemic lupus erythematosus; IgG: immunoglobulin G; IgM: immunoglobulin M; CSF: cerebrospinal fluid; D/D: differential diagnosis; OCT: optical coherence tomography.

S. No.	Year	Authors	Age/Sex	Initial Diagnosis	Diagnosis Confirmation	Treatment	Outcome
1	2003	Yamamoto et al. [28]	47/F	ARN	Vitreous PCR - positive for *T. gondii*, chorioretinal biopsy - necrotic retina with active *T. gondii* bradyzoites	Pyrimethamine, sulfadiazine, folinic acid supplementation	Improved
2	2019	Furuya et al. [18]	43/F	CMV, neuropsychiatric SLE	Elevated *T. gondii* IgG and IgM titres in serum and CSF, positive *T. gondii* PCR in CSF, positive *T. gondii* immunostaining and PCR on transbronchial lung biopsy specimen	Pyrimethamine + sulfadiazine	Improved
3	2024	Yang and Li [29]	44/F	SLE associated retinochoroiditis	Serological tests - positive Toxo IgM, aqueous humor analysis - elevated Goldmann Witmer coefficient	Pyrimethamine + sulfadiazine	Improved
4	2024	Singh et al. [30]	60/M	D/D – Viral retinitis, tubercular chorioretinitis, Toxoplasma retinochoroiditis	Positive Toxo IgM and IgG on serological tests, OCT - disorganisation of retinal layers and hyperreflectivity of retinal layers	Intravitreal clindamycin, dexamethasone injection, systemic trimethoprim and sulfamethoxazole, oral steroids	Improved

In the case reported by Singh et al., the final diagnosis was made based on positive Toxoplasma IgG and IgM titres on serological examination and the classic OCT picture [30]. In the present case, the diagnosis of ocular toxoplasmosis was established on the basis of unilateral active retinochoroiditis with vitritis, in the absence of a pre-existing pigmented chorioretinal scar, which made the presentation atypical. Optical coherence tomography demonstrated a distortion of the retinal and choroidal architecture along with retrohyaloid hyperreflective deposits, supporting the diagnosis. Serological evaluation revealed positive *T. gondii* IgG with negative IgM, suggestive of prior exposure with likely reactivation. The Standardized Uveitis Nomenclature (SUN) working group classification criteria for toxoplasma retinochoroiditis require focal or paucifocal necrotizing retinitis combined with either a characteristic clinical picture (e.g., retinitis adjacent to a pigmented scar) or positive *T. gondii* PCR/laboratory testing from an ocular specimen [31]. Our case differed in lacking a pre-existing toxoplasmic scar, thereby representing an atypical presentation. In this setting, multimodal imaging was critical in establishing the diagnosis.

At presentation to us, the patient was receiving a maintenance dose of hydroxychloroquine 200 mg on alternate days. However, the absence of overt systemic activity in SLE does not necessarily imply restoration of normal immune function, as persistent intrinsic immune dysregulation - including complement abnormalities, impaired cellular immune responses, and defective pathogen clearance - may continue despite clinical quiescence [32-35]. Interestingly, despite this underlying immune vulnerability, the ocular presentation was more consistent with the pattern typically described in immunocompetent individuals, with unilateral involvement and a solitary focus of active retinochoroiditis with vitritis, rather than the multifocal, bilateral, or diffuse necrotizing retinitis more often reported in markedly immunocompromised hosts [20-22]. This suggests that there is persistent immune modulation inherent to SLE, which may have predisposed to infection without altering its classical ocular phenotype. The subsequent development of systemic flare-up, followed by herpes zoster, further supports the presence of ongoing subclinical immune dysregulation at the time of ocular infection, indicating that intrinsic immune abnormalities in SLE may persist even during periods of apparent clinical stability.

Seroprevalence of *T. gondii* infection is relatively high across the globe. Toxoplasma-specific IgG is invariably positive in patients with ocular toxoplasmosis; but it is also present in infected individuals without any ocular involvement. Hence, Toxoplasma-specific IgG has little diagnostic value [36,37]. *T. gondii*-specific IgM antibodies may be identified in the serum of certain patients, potentially indicating a recent infection. However, in acute cases, equivocal or even positive IgM results may have limited diagnostic value. Additionally, the parasite has been detected in the peripheral blood both of patients with ocular toxoplasmosis and in control individuals [38]. Consequently, the presence of the parasite or certain antibodies in peripheral blood does not prove ocular involvement [39].

In the present case, IgG for Toxoplasma, CMV, and Rubella were positive and IgM for all three were negative. IgG antibodies reflect immunological memory (previous exposure, latent infection or immunity), while IgM antibodies are markers of recent infection [40]. A national serological survey in India reported that the seroprevalence of Toxoplasma ranged from 9.4% to 19.7% in the North Indian population [41], which reflects a moderate level of exposure to this pathogen in the general population. Studies have shown the prevalence of anti-Rubella antibodies to be 82.6% in North India [42] and 80% in the state of Uttarakhand [43]. This has been attributed to the extensive vaccination program in the country. The seroprevalence of CMV in India was reported to be between 80% and 95% [44,45], which has been attributed to widespread prior exposure to the virus. So, in general, the seroprevalence of Rubella and CMV IgG antibodies is higher in the population as compared to Toxoplasma.

In a considerable percentage of patients (21%), there is a clinically notable time lapse (up to two to three weeks) between the appearance of symptoms and the onset of local antibody production [46]. This delay has been observed in both primary and recurrent cases of ocular toxoplasmosis [39,47]. In our case, enzyme-linked immunosorbent assay (ELISA) for Toxoplasma was done within eight days of the onset of symptoms, so that might be the reason for a negative IgM test.

Garweg et al., in their article about the diagnostic approach to ocular toxoplasmosis [39], concluded that depending on the time of sampling, laboratory testing may support the clinical diagnosis of ocular toxoplasmosis in 60%-85% of cases. When the retinal lesions are characteristic of toxoplasma retinochoroiditis, accompanied by positive *T. gondii*-specific IgG and negative IgM in serum, and there is a favorable response to appropriate anti-Toxoplasma therapy, most authorities consider this to represent a reactivated form of ocular toxoplasmosis [39].

The rupture of intraretinal cysts can cause reactivations at any point after the primary infection, triggering a rapid localized immune reaction. It is important to emphasize that lack of posterior segment scarring is not pathognomonic of a recent infection with *T. gondii* and does not rule out the possibility of congenital disease [48].

In immunocompetent individuals, amplification of Toxoplasma DNA in aqueous humor samples is achievable only in about 30%-40% of the clinically diagnosed cases [49-51], whereas the detection rate rises to around 75% in immunocompromised patients [50,51]. A limited amount of the sample available for analysis, low parasitic load in the aqueous humor (even in situations of acute infection), and/or an early degradation of its DNA may contribute to the relatively low rates of DNA amplification [47,52]. Furthermore, disruption of the blood-retinal barrier permits an influx of serum antibodies into the intraocular compartment, where their high concentrations can mask localized antibody production within the eye [53]. Amplification of parasitic DNA from vitreous samples has been reported in up to 50% of immunocompetent patients with clinically diagnosed ocular toxoplasmosis [54]. Nevertheless, a laboratory confirmation remains elusive in nearly 15%-40% of patients [39]. Only in severe atypical or complicated cases and cases that fail to respond to anti-Toxoplasma therapy, the withdrawal of ocular fluid samples is justified [39].

Therefore, accurate diagnosis primarily relies on recognizing the spectrum of clinical manifestations [14,55]. Ocular toxoplasmosis typically manifests as a whitish focus of necrotizing retinochoroiditis [14,55] with associated retinal and choroidal necrosis and destruction of the surrounding tissues. It is generally accompanied by vitritis and even with anterior uveitis [14]. As the active retinochoroidal lesion resolves, it usually leads to an atrophic retinochoroidal scar, with healing occurring from the periphery to the centre of the lesion [14]. The same classic clinical picture, at presentation and during the healing stage was seen in the present case.

Agarwal et al. [56] have described in detail multimodal imaging in ocular toxoplasmosis - OCT shows the presence of retinal distortion, hyperreflectivity, and thickening along with retrohyaloid hyperreflective spots and the presence of hyperreflective round deposits along the posterior hyaloid [56]. Additionally, disruption of choroidal architecture with focal choroidal thickening beneath the areas of necrotizing retinitis is significantly associated with toxoplasmosis [56-58]. Our OCT picture showed both - retinal and choroidal distortion in the area of the lesion - and hyperreflective round deposits along the posterior hyaloid, similar to that described in these publications.

## Conclusions

This case report aims to emphasize that toxoplasmosis can occur in immunosuppressed patients like those with SLE, even when there is no clinically apparent systemic activity, reflecting the persistent intrinsic immune dysregulation associated with SLE. Therefore, clinicians should maintain a high index of suspicion for this opportunistic infection. Ocular toxoplasmosis may present with markedly atypical features in the setting of immunosuppression and is often prone to misdiagnosis. In contrast to the previously reported cases of ocular toxoplasmosis in SLE, which were associated with atypical or fulminant ocular presentations and diagnostic uncertainty, our case presented with a classic clinical picture of unilateral solitary retinochoroiditis with vitritis. Multiple aspects of the laboratory-based diagnosis of ocular toxoplasmosis are inadequately understood. In this context, the diagnosis is frequently based on characteristic clinical findings supported by multimodal ocular imaging, particularly when corroborated by a favourable response to anti-toxoplasma therapy.

Toxoplasmosis in a case of SLE can potentially have a lethal outcome as it frequently involves the brain. A prompt and accurate diagnosis of ocular toxoplasmosis and timely initiation of appropriate treatment may help in saving both vision and life.
